# Divergent Astrovirus Associated with Neurologic Disease in Cattle

**DOI:** 10.3201/eid1909.130682

**Published:** 2013-09

**Authors:** Linlin Li, Santiago Diab, Sabrina McGraw, Bradd Barr, Ryan Traslavina, Robert Higgins, Tom Talbot, Pat Blanchard, Guillermo Rimoldi, Elizabeth Fahsbender, Brady Page, Tung Gia Phan, Chunlin Wang, Xutao Deng, Patricia Pesavento, Eric Delwart

**Affiliations:** Blood Systems Research Institute, San Francisco, California, USA (L. Li, B. Page, T.G. Phan, X. Deng, E. Delwart);; University of California San Francisco, San Francisco (L. Li, T.G. Phan, X. Deng, E. Delwart);; University of California Davis, Davis, California, USA (S. Diab, S. McGraw, B. Barr, R. Traslavina, R. Higgins, P. Blanchard, G. Rimoldi, P. Pesavento);; California Animal Health and Food Safety Laboratory, Davis (S. Diab, B. Barr, P. Blanchard, G. Rimoldi);; Bishop Veterinary Hospital Inc., Bishop, California, USA (T. Talbot);; University of South Florida, St. Petersburg, Florida, USA (E. Fahsbender);; Stanford Genome Technology Center, Stanford, California, USA (C. Wang)

**Keywords:** Astrovirus, brain, neurologic disease, bovine, cattle, next-generation sequencing, in situ hybridization, viruses

## Abstract

Using viral metagenomics of brain tissue from a young adult crossbreed steer with acute onset of neurologic disease, we sequenced the complete genome of a novel astrovirus (BoAstV-NeuroS1) that was phylogenetically related to an ovine astrovirus. In a retrospective analysis of 32 cases of bovine encephalitides of unknown etiology, 3 other infected animals were detected by using PCR and in situ hybridization for viral RNA. Viral RNA was restricted to the nervous system and detected in the cytoplasm of affected neurons within the spinal cord, brainstem, and cerebellum. Microscopically, the lesions were of widespread neuronal necrosis, microgliosis, and perivascular cuffing preferentially distributed in gray matter and most severe in the cerebellum and brainstem, with increasing intensity caudally down the spinal cord. These results suggest that infection with BoAstV-NeuroS1 is a potential cause of neurologic disease in cattle.

Astroviruses are small, nonenveloped, positive single-stranded RNA viruses with a genome of 6.4–7.3 kb. The family *Astroviridae* comprises 2 genera, *Mamastrovirus* and *Avastrovirus*, known to infect mammals and birds, respectively. Since the first description of human astrovirus (HAstV) in children with diarrhea in 1975 (*1*), a wide variety of astroviruses have been reported in multiple animals including humans, cattle, pigs, sheep, minks, dogs, cats, mice, sea lions, whales, chickens, and turkeys (*2*).

Enteric astroviruses are transmitted through the fecal–oral route, and regardless of species, most infections are asymptomatic. In humans, the prevalence of exposure is very high, and astrovirus infection is a major cause of acute enteritis in infants (*3*). Clinical disease also can affect elderly and immunocompromised persons. In these persons, the clinical course of infection is acute, with 2–4 days of watery diarrhea and, less commonly, vomiting, headache, fever, abdominal pains, and anorexia (*4*).

Astroviruses have been implicated twice in central nervous system (CNS) disease (*5*,*6*). One study demonstrated an HAstV-PS, which is distinct from the original HAstV and closely related to astrovirus HMO-C (AstV-HMO-C) and HAstV-VA1) (*7*,*8*) in the brain tissue of a 15-year-old boy with X-linked agammaglobulinemia who had encephalitis. HAstV-PS was the only virus detected, and astrocyte infection was confirmed by anticapsid antibody staining (*6*). Serologic evidence of exposure to the closely related AstV-HMO-C was found in 36% of 5–10-year-old children in the United States, which reflects a common childhood infection and indicates that the encephalitis in this child was a likely consequence of his immunodeficiency (*9*). In an outbreak of so-called “neurological shaking disease” in mink, an astrovirus (Mink AstV-SMS) was detected in the brain tissues of multiple naturally and experimentally infected animals showing neurologic signs, including shaking and ataxia (*5*).

Cattle with neurologic signs are vigilantly screened to keep the food chain free of zoonotic pathogens, such as rabies virus, *Salmonella* spp., *Listeria monocytogenes*, *Chlamydia* spp., and the prion agent of bovine spongiform encephalopathy (BSE). In particular, BSE has become a major public health concern after recognition of the association between BSE and prion-associated disease in humans. Therefore, early and rapid recognition of the cause of neurologic disease is vital to the safety of the food chain. Etiologic diagnosis of CNS disease in cattle requires substantial effort; is costly; and usually presents a challenge because of the large number of pathogens or problems that can cause neurologic disease, including viruses, bacteria, parasites, prions, toxins, and metabolic disorders. Pathogens known to cause CNS disease in cattle include bovine herpesvirus 1 and 5 (BoHV-1 and BoHV-5), lyssavirus (rabies), ovine herpesvirus 2, *L. monocytogenes*, *Histophilus somni*, *Escherichia coli*, *Salmonella* spp., *Chlamydia* spp., *Neospora caninum*, amoebas, and prions (*10*,*11*).

Brain tissue from a yearling steer with an encephalomyelitis and ganglioneuritis of unknown origin was analyzed by using viral metagenomics, which showed a divergent astrovirus distantly related to an ovine astrovirus. By retrospective analysis, this bovine astrovirus associated with neurologic symptoms (BoAstV-NeuroS1) was detected in the brains of 3 of 32 other cattle with encephalitides of undetermined etiology. Virus was detected by RNA by in situ hybridization within neurons in the brainstem, cerebellum, and/or spinal cord in all PCR positive samples from the 4 animals in this study.

## Materials and Methods

### Sample Preparation and Next-Generation Sequencing

To search for potential viral etiologic agents, we performed an unbiased metagenomic analysis (*12*). Viral nucleic acids were enriched from fresh-frozen brain tissue samples (≈25 mg) by tissue homogenization, filtration, and nuclease treatment, and a library of randomly amplified PCR products from viral RNA and DNA was prepared by using a ScriptSeq version 2 RNA-Seq library preparation kit (Epicenter, Madison, WI, USA) and sequenced on the MiSeq Illumina platform (Illumina, San Diego, CA, USA) (*13*).

### Bioinformatics Analysis

Paired-end reads of 250 bp generated by MiSeq were debarcoded by using vendor software from Illumina. An in-house analysis pipeline running on a 32-node Linux cluster was developed to process the data. Clonal reads were removed, and low sequencing quality tails were trimmed by using Phred quality score 10 as the threshold. Adaptors were trimmed by using the default parameters of VecScreen, which is NCBI BLASTn (*14*) with specialized parameters designed for adaptor removal. The cleaned reads were assembled de novo by using SOAPdenovo2 (*15*). The assembled contigs, along with singlets, were aligned to an in-house viral proteome database by using BLASTx. The significant hits to virus were then aligned to an in-house nonvirus–nonredundant universal proteome database by using BLASTx. Hits with more significant adjusted E-value to nonviral than to viral sequences were removed.

### Genome Sequencing and Phylogenetic Analyses

The presence of astrovirus genomic sequences assembled from next-generation sequencing reads were confirmed by PCR and Sanger sequencing. By connecting gaps between sequenced viral fragments, and amplifying the 5′ and 3′ end sequences by using 5′ and 3′ rapid amplification of cDNA ends (RACE) (*16*,*17*), the complete genome of the new astrovirus was obtained. Phylogenetic analyses based on aligned amino acid sequences from full-length protease, RNA-dependent RNA polymerase (RdRp), and capsid proteins were generated by the neighbor-joining method in MEGA 4 (*18*) by using amino acid p-distances with 1,000 bootstrap replicates. Maximum parsimony and maximum likelihood methods were conducted to confirm the topology of the neighbor-joining tree.

### Retrospective Search for BoAstV-NeuroS1

We selected 32 cases of histologically confirmed bovine encephalitis, each of undetermined etiology, from the archives (2003–2013) of the Veterinary Medical Teaching Hospital (Davis, CA, USA) and at the Davis, Tulare, and San Bernardino branches of the University of California, Davis–California Animal Health and Food Safety laboratory system. All animals had a histologic diagnosis of either nonsuppurative or pleocellular encephalitis, encephalomyelitis, or meningoencephalitis. Histology was reviewed, and nucleic acids were extracted from selected sections of formalin-fixed, paraffin-embedded affected brain tissue by Agencourt Formapure Kit (Beckman Coulter, Atlanta, GA, USA) (*19*).

Reverse transcription nested PCR was used to detect BoAstV-NeuroS1 in nucleic acid extracts from formalin-fixed, paraffin-embedded tissue wax scrolls. cDNA was generated with Superscript III (Invitrogen, Carlsbad, CA, USA) and random hexamer. The oligonucleotide primer sets used were as follows: AstV-sF1: 5′-ACCGCCTTTCTGATGATGTGC-3′; AstV-sR1: 5′-CTCATCAACAACCTGCCAAAT-3′; AstV-sF2: 5′-GACTCTGAGGGTCAAATAACC-3′; AstV-sR2: 5′-GCCAAATGGTTTCTCCAACAG-3′ (capsid gene region, ≈150-bp amplicon); AstV-sF3: 5′-ACCGCCTTTCTGATGATGTGC-3′; AstV-sR3: 5′-CTCATCAACAACCTGCCAAAT-3′; AstV-sF4: 5′-GACTCTGAGGGTCAAATAACC-3′; and AstV-sR4: 5′-GCCAAATGGTTTCTCCAACAG-3′ (protease gene region, ≈150-bp amplicon). The PCR conditions were 95°C for 2 min, followed by 39 cycles of 95°C for 15 sec, 55°C for 15 sec, and 72°C for 15 sec, and a final extension at 72°C for 10 min. PCR products were checked by 2% agarose gel electrophoresis, and amplicons of the appropriate size were confirmed by sequencing. PCR-positive and -negative (control) samples were concurrently used for in situ hybridization analyses.

### Testing of Affected Steer

The animal was submitted to the California Animal Health and Food Safety Laboratory in Davis, California, for euthanasia and postmortem examination. PCR tests for BoHV-1, bovine viral diarrhea virus (BVDV), West Nile virus, bluetongue virus, epizootic hemorrhagic disease virus, and ovine herpesvirus 2 were negative. BVDV, BoHV-1, *L. monocytogenes*, and *Neospora* spp. were also negative by immunohistochemical examination of sections. Samples were negative for rabies virus according to fluorescent antibody test. Virus isolation, done independently at Cornell University (Ithaca, NY, USA) and at the California Health and Food Safety Laboratory (Davis, CA, USA) from a brain pool was negative. The tissue sample was placed onto bovine turbinate and swine kidney primary cell cultures. Two passages were made. At the end of the second passage, the cells were stained with a polyvalent viral antiserum, a pseudorabies antiserum, and bovine enterovirus 1–7 antiserum in an indirect immunofluorescence assay. In the sentinel animal, serologic test results for BoHV-1 were negative and for BVDV types 1 and 2 were positive at a titer of 512. *Ostertagia* spp. were detected in the abomasal contents, and *Trichostrongyle* spp. eggs and *Coccidia* oocysts were found in feces. Hepatic levels of copper (5.8 ppm) and selenium (0.13 ppm) were interpreted as being low.

### Histology and In Situ Hybridization

Brains from all animals and various segments of the spinal cord from 3 animals were immersion-fixed in 10% buffered formalin, pH 7.2, for at least 48 h. Transverse sections of the brain, including cerebral cortex and corona radiata, basal nuclei, thalamus, midbrain, medulla oblongata, cerebellar peduncles, cerebellum and brainstem in all animals; the full length of the spinal cord in animals 1 and 2 (including cervical dorsal root ganglia in animal 1); and cervical cord segments only in animal 3 were processed by standard histologic techniques by using 4-µm thick sections stained with hematoxylin and eosin.

Colorimetric in situ hybridization was performed on 4-μm thick tissue sections, mounted on 3-aminopropyltriethoxylane–coated slides (Fisher Scientific, Freemont, CA, USA) by using a 28-nt oligomer probe (5′-ACATGGCTGTAAGCATTGGTGTGAAGTA-3′) complementary to the capsid region of BoAstV-NeuroS1 and 3′-labeled with digoxigenin-II-dideoxy undine (Eurofins MWG Operon (Huntsville, AL, USA). Tissue sections were deparaffinized with d-limonene (CitriSolv; Fisher Scientific) and digested by incubation with 0.25% pepsin in Tris-buffered saline (pH 2.0) at 37°C for 10 min, followed by a 5-min incubation at 105°C to stop enzymatic activity. Nucleic acid denaturation was achieved by incubation in formamide (100%) at 105°C for 5 min. Hybridization was performed at 37°C in a 10-µmol solution of the digoxigenin-labeled probe in hybridization buffer (22.5% deionized formamide, 7.5% chondroitin sulfate, 5× saline sodium citrate, 0.25% blocking reagent, and 50 mmol phosphate buffer). Sections were incubated in a digoxigenin antibody solution (500:1 dilution) containing 2.5 mL buffer 1 (100 mM Tris, 150 mM NaCl, 0.3% Triton X-100, 1% goat serum, pH 7.5) with 5 μL of antidigoxigenin Fab fragments conjugated with alkaline phosphatase (750 U/mL) (Roche, Mannheim, Germany). Sections were then washed and developed according to manufacturer instructions before counterstaining with 1% fast-green FCF for 5 min. After counterstaining, slides were coated with aqueous mounting media (ImmunoHistoMount, Immunobioscience, Mukilteo, WA, USA) and covered with SHUR/Mount mounting medium (Triangle Biomedical Sciences, Durham, NC, USA). There was no observed hybridization in replicate tissue sections incubated with another, unrelated digoxigenin-labeled probe with similar guanine-cytosine content, and brain tissues from astrovirus-negative cattle exhibited no staining.

### Electron Microscopy

Selected pieces of wax-embedded spinal cord tissue from the sentinel animal were extracted and postfixed in 2.0% glutaraldehyde and then routinely processed and embedded in epoxy resin (Eponate12 kit; Ted Pella, Redding, CA, USA). Selected thick sections were stained with toluidine blue. Ultrathin sections were examined by using a Zeiss (Göttingen, Germany) 906E transmission electron microscope.

## Results

### Results of Metagenomic and Phylogenetic Analysis of BoAstV-NeuroS1 in Sentinel Animal

A 168-kg crossbreed yearling steer originating from Mexico was found on a property in northern California in lateral recumbency, with opisthotonus and limbs in extensor rigidity. After deep sequencing of enriched viral particles from homogenized diseased brain tissue, we identified 170 sequence reads related to astroviruses using BLASTx (E-score <10-5), which could be assembled into 17 contigs covering ≈45% of the viral genome.The complete genome of the new astrovirus (GenBank accession no. KF233994) was then generated by linking fragments by PCR and by using 5′ and 3′ RACE to yield a genome provisionally named bovine astrovirus NeuroS1 (BoAstV-NeuroS1).

The resulting genome was 6,471 nt long, with a GC content of 48%. As typical mamastroviruses, BoAstV-NeuroS1 had 3 putative open reading frames (ORFs), encoding the protease with ORF1a (860 aa), RdRp with ORF1b (525 aa), and capsid protein with ORF2 (757 aa). An expected ribosomal frame-shift signal was found in the ORF1a/1b overlap region that consisted of the heptameric AAAAAAC sequence, followed by a potential 20-nt pseudo-knot sequence. The conserved protease motifs and RdRp motifs, such as the potential conserved proteolytic cleavage site (VHL/TNT) and the characteristic YGDD motif, were present. The amino-terminal half of the capsid protein was more conserved than the carboxy-terminal region.

**Figure 1:**
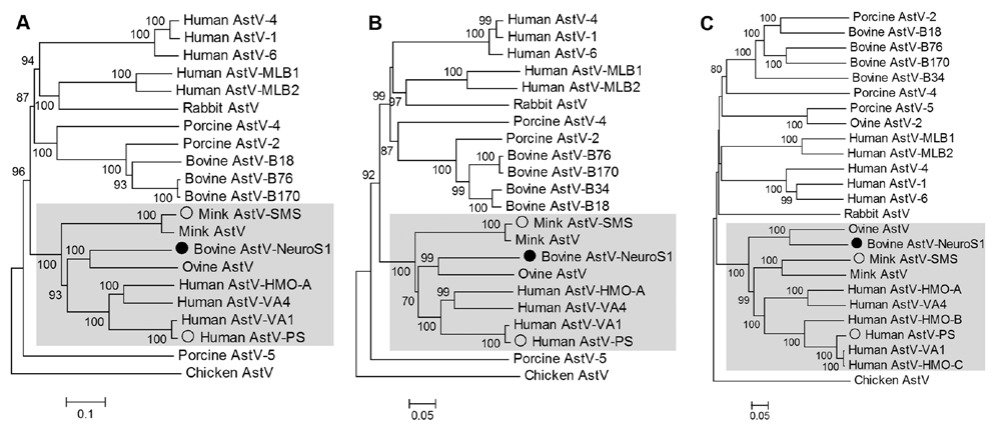
Phylogenetic tree based on aligned amino acid sequences of the full length of the protease (open reading frame [ORF] 1a) (A), RNA-dependent RNA polymerase (ORF1b) (B), and capsid (ORF2) region (C) of representative astrovirus (AstV) species. BoAstV-NeuroS1 labeled with filled circle. AstVs with neurotropic potential labeled by empty circles. Clade containing these 3 viruses is shaded. Scale bar indicatesestimated protein sequence phylogenetic distance. GenBank accession numbers for astrovirus used in the analysis are as follows: bovine BoAstV-NeuroS1 (KF233994), human AstV-1 (JN887820), human AstV-4 (DQ344027), human AstV-6 (HM237363), human AstV MLB1 (JQ086552), human AstV MLB2 (NC_016155), human AstV-VA1 (FJ973620), human AstV-VA4 (JX857869), human AstV-HMO-A (NC_013443), human AstV-HMO-B (GQ415661), human AstV-HMO-C (GQ415662), human AstV-PS (GQ891990), ovine AstV (NC_002469), ovine AstV-2 (JN592482), bovine AstV B18 (HQ916313), bovine AstV-B34 (HQ916315), bovine AstV-B76 (HQ916316), bovine AstV-B170 (HQ916314), porcine AstV-2 (JF713712), porcine AstV-5 (JF713711), mink AstV (AY179509), mink AstV-SMS (GU985458), rabbit AstV (JF729316), and chicken AstV (JF414802).

To determine the genetic relationship between BoAstV-NeuroS1 and other astroviruses, we performed sequence alignments of the protease (ORF1a), RdRp (ORF1b), and capsid (ORF2). BoAstV-NeuroS1 shared the highest identity of 56%, 70%, and 66% aa similarity with the ORF1a, ORF1b, and ORF2 encoded proteins of ovine astrovirus, its closest relative. Phylogenetic analysis was performed, and neighbor-joining trees were generated (Figure 1). All 3 trees confirmed that BoAstV-NeuroS1 was most closely related to the ovine astrovirus prototype, which was identified in 1977 (*20*) and sequenced in 2003 (*21*), but was phylogenetically far from the recently reported ovine AsttV2 (*22*), and other known BastVs.

### Retrospective Study

For 32 animals, formalin-fixed, paraffin-embedded tissues of bovine brain or spinal cord (1 or 2 tissue sections per animal) were tested for BoAstV-NeuroS1 by specific reverse transcription nested PCR, and samples from 3 (9.4%) animals (animals 2–4) were positive. Samples from these same animals also were positive by in situ hybridization. Animal 2 was a 178-kg, polled Hereford heifer from northern California that was found recumbent with intermittent seizures, unable to move the hind legs, and lacking hind limb pain withdrawal reflex. Animal 3 was a euthanized 3-year-old Angus cow that on the farm was unable to rise and exhibited occasional star gazing and unresponsiveness to thiamin therapy. Animal 4 was a euthanized 3-year-old Holstein cow with a history of circling and blindness. The laboratory diagnostic workup for CNS pathogens in these 3 additional animals was not consistent but included negative test results for BVDV, BoHV-1, and rabies virus. Culture for *L. monocytogenes* in animals 3 and 4 was negative; for animals 2 and 4, *Neospora caninum*, *Sarcocystis* spp., BSE, and pseudorabies histologic lesions were negative by immunohistochemistry; and immunohistochemical analysis results for West Nile virus, *Chlamydia* spp. and *Toxoplasma* spp. in animal 3 were negative.

### Histology, In Situ Hybridization, and Ultrastructure

**Figure 2:**
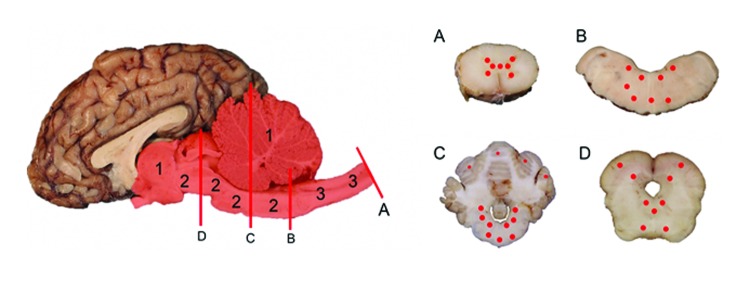
Yearling steer with encephalomyelitis. Midsagittal section of brain and multiple transverse sections of cerebellum, brainstem, and spinal cord depicting the location and severity of microscopic lesions. Midsagittal section of the brain: red highlight indicates areas of the central nervous system affected, numbers indicate severity of the lesions (1 = least severe; 2 = more severe; 3 = most severe), and red lines (A, B, C, D) indicate the levels where transverse sections were cut. A, spinal cord. B, medulla oblongata. C, cerebellum and cerebellar peduncles. D, midbrain (superior colliculus). Cross-sections of brainstem, cerebellum, and spinal cord: red dots indicate sites and relative intensity of microscopic lesions.

The anatomic pattern of lesion distribution was remarkably consistent and unusual in all 4 animals. The meningoencephalomyelitis was largely confined to gray matter in the brain and spinal cord, with most severe lesions in the cerebellar folia and brainstem and throughout all segments of the spinal cord gray matter. Rostrally, there was minimal involvement in the midbrain, thalamus, and basal nuclei, but the cerebral cortex and underlying corona radiata were devoid of inflammatory cell infiltrates (Figure 2, Appendix). There was a nonsuppurative ganglioneuritis in the 1 cervical dorsal root ganglion examined.

**Figure 3:**
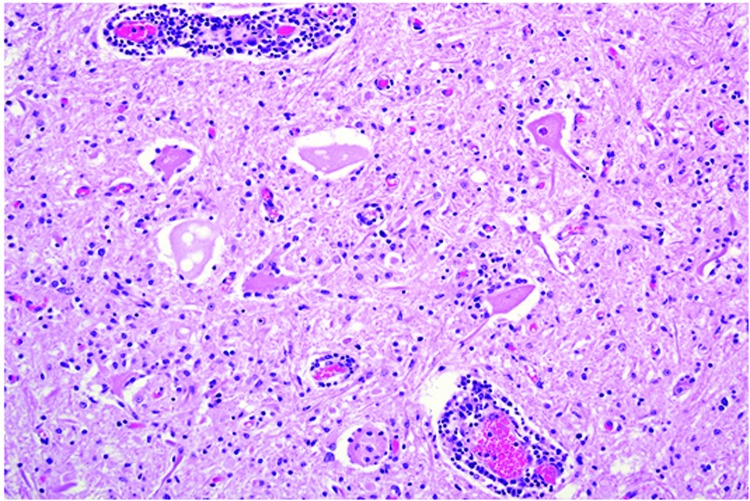
Spinal cord at the L5 segment of a heifer with encephalomyelitis (animal 2). Note the nonsuppurative encephalomyelitis with lymphocytic perivascular cuffs, neuronal degeneration and necrosis, spheroids from necrotic neurons, and neuronophagia with widespread microgliosis. Hematoxylin and eosin stain. Original magnification ×400.

**Figure 4:**
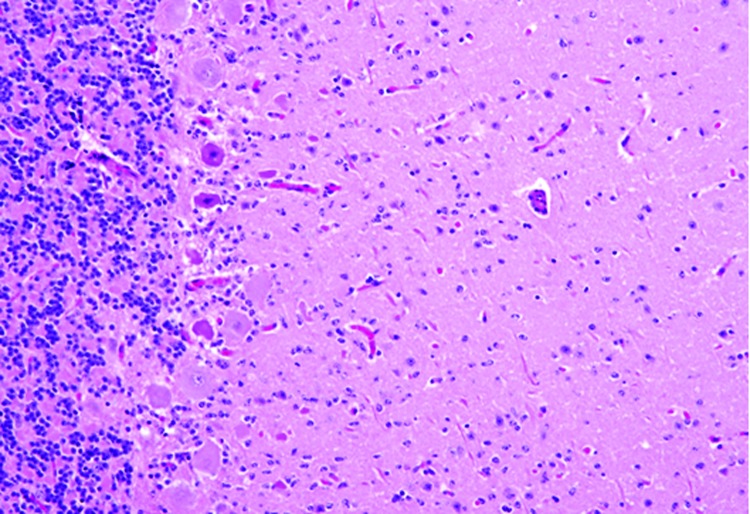
Cerebellum of a yearling steer with encephalomyelitis (animal 1). Note the selective extensive acute necrosis and degeneration of Purkinje cells. Numerous necrotic dendritic spheroids in the molecular layer with a cellular proliferation of Bergmann glia and of microgliosis. Hematoxylin and eosin stain. Original magnification ×400.

**Figure 5:**
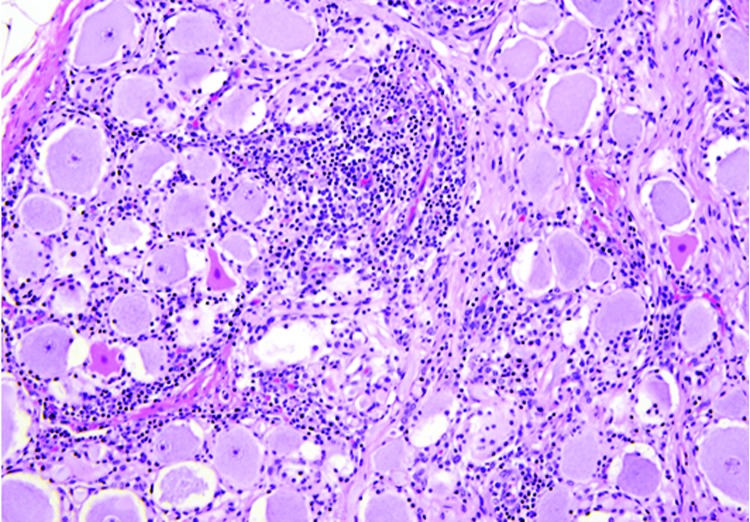
Dorsal root ganglion of a heifer with encephalomyelitis (animal 2). Multifocal marked interstitial lymphocyte, macrophage, and plasma cell infiltrates with multifocal neuronal degeneration and necrosis can be seen. Hematoxylin and eosin stain. Original magnification ×400.

Microscopically in all animals, the lesions were pathognomonic for a highly neurotropic viral encephalomyelitis and included moderate to marked lymphocytic cell perivascular cuffing and neuronal and ganglionic degeneration and necrosis, with microgliosis but minimal meningitis, especially in the dorsal and ventral horns of the spinal cord gray matter and multifocally in the medulla oblongata, cerebellar peduncles, and midbrain (Figure 3). Degenerating or necrotic neurons were variously swollen and hypereosinophilic and shrunken with angular borders and/or had faded, pale, or eosinophilic cytoplasm sometimes with vacuolation or central pallor. Nuclei of affected neurons remained central but were variably pyknotic, karyorrhectic, or rarely karyomegalic with absent or dispersed chromatin. Neuronophagia of these neurons by microgliosis was not a common feature and appeared delayed but was occasionally seen in individual neurons with an eosinophilic irregular granular content, especially in the gray matter of the spinal cord. Eosinophilic swollen axonal and dendritic spheroids accompanied this neuronal necrosis. The cerebellar folia also had widespread lesions, with a predilection for Purkinje cell necrosis and loss, with characteristic Bergmann glial and microglial proliferation and dendritic spheroids in the overlying layer; however, axonal torpedoes were rarely found (Figure 4). Minimal lesions occurred as far rostrally as the thalamus, and basal nuclei and were limited to mild lymphocytic and plasma cell perivascular cuffing, with scattered microgliosis and, rarely, neuronal necrosis. The cerebral cortex and underlying white matter was surprisingly free of inflammatory lesions. In addition, a dorsal root ganglion of animal 2 showed lymphocytic ganglioneuritis with occasional characteristic neuronal degeneration and necrosis (Figure 5). Throughout the length of the spinal cord in animal 2, there was a bilateral asymmetric polyneuritis restricted to the dorsal nerve roots at most segmental levels.

**Figure 6:**
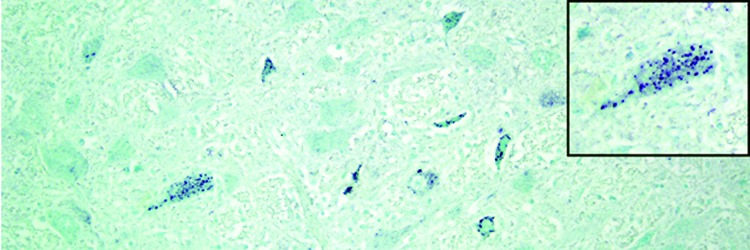
Medulla oblongata of a heifer with encephalomyelitis (animal 2). Punctate cytoplasmic staining (green) in multiple neurons within a nucleus can be seen; inset shows a higher magnification of a positive brainstem neuron. In situ hybridization for viral RNA. Original magnification ×400.

**Figure 7:**
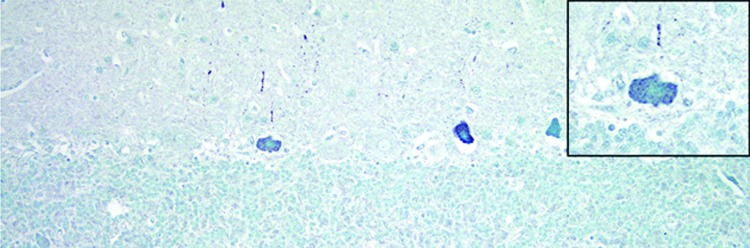
Cerebellum of a yearling steer with encephalomyelitis (animal 1). Punctate to diffuse positive (green) staining of Purkinje cells cytoplasm and dendritic processes can be seen; inset shows a higher magnification of a positive Purkinje cell. In situ hybridization for viral RNA. Original magnification ×400.

**Figure 8:**
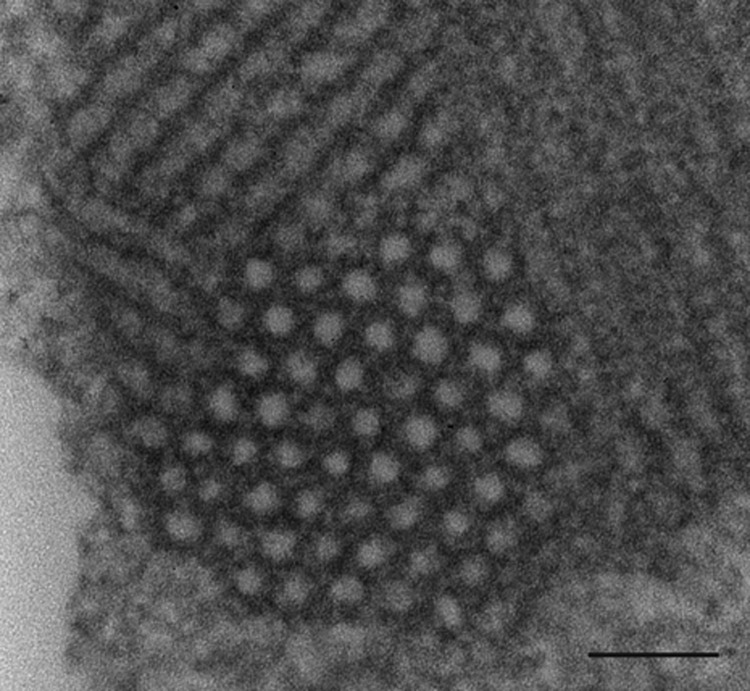
Transmission electron microscopic image of necrotic neuron in the lumbar region of a heifer with encephalomyelitis. Intracytoplasmic paracrystalline array of 27–28-nm diameter viral-like particles. Scale bar indicates 100 nm.

With in situ hybridization, many affected neurons had variably distinct, punctate to diffuse cytoplasmic staining throughout the cytoplasm or occasionally eccentrically located (Figure 6). In the cerebellar folia, degenerative and necrotic Purkinje cells with their associated necrotic dentritic spheroids in the molecular layer were positive for BoAstV-NeuroS1 (Figure 7). Ultrastructural analysis of the spinal cord from the sentinel steer showed paracrystalline, stacked arrays of empty viral-like particles within the cytoplasm of neurons (Figure 8). The diameter of individual particles was ≈27.5 nm.

## Discussion

We report the genomic characterization of a novel astrovirus (BoAstV-NeuroS1) in the brain tissue of 4 cattle from ranches in California with a clinical neurologic disorder characterized histologically as a neurotropic meningoencephalomyelitis and ganglioneuritis. The virus was initially identified by viral metagenomics, and its presence was subsequently confirmed retrospectively in the CNS of 3 other cattle that had bovine encephalomyelitis of unknown etiology by PCR and in situ hybridization for viral RNA. Also, ultrastructure studies in 1 animal demonstrated intracytoplasmic particles in degenerating neurons tentatively considered morphologically compatible with an astrovirus. Our attempts to recover this virus in tissue culture have not been successful, and no concurrent intestinal infection was detected.

Other astroviruses have been identified and associated with neurologic signs in an immunodeficient child and in minks with a shaking syndrome in farm outbreaks reported in Denmark, Sweden, and Finland (*5**,**6*). BoAstV-NeuroS1 infection shares neuropathologic features with the disease in mink. Both diseases have a seemingly identical anatomic distribution and type of lesions, and both occur with neurologic symptoms generally as isolated cases in mixed-breed animals.

BoAstV-NeuroS1 is now the third separate astrovirus species detected in brain tissue that has been associated with neurologic disease. It may be relevant that these 3 astrovirus species associated with neurologic symptoms (HAstV-PS, mink AstV-SMS, and BoAstV-NeuroS1) fall into the same genetic clade of astroviruses, together with viruses of other species so far not associated with disease (Figure 1).

Bovine neurologic diseases can be caused by bacteria, parasites, viruses, toxins, or nutritional disturbances (*10*,*11*,*23*). Common infectious agents causing neurologic disease in cattle are regionally variable but include *Clostridium botulinum*, *Clostridium tetani*, *L. monocytogenes, Histophilus somni, Babesia bovis,* BSE prion, and BoHV-1 and -5. Infection with BoAstV-NeuroS1 is a new addition to the differential diagnosis of neurologic disorders in cattle, and constituted ≈9% of the undiagnosed cases of encephalitis in our retrospective study. This finding probably underestimates the prevalence of BoAstV-NeuroS1 disease because detection in this retrospective study was limited to formalin-fixed, paraffin-embedded tissues from a single region of the brain. Our preliminary microscopic and in situ hybridization results suggest that the best target for virus detection would be the spinal cord. Rapid diagnosis of astrovirus RNA by PCR or in situ hybridization in the brain or spinal cord of cattle with neurologic signs may enable more rapid exclusion of infection with the BSE prion. The involvement of the cerebellum is distinctive and rare and has been reported only with the still enigmatic European sporadic bovine encephalitis. However, the neurotropic lesions in that disease also intensively involve the hippocampus. Arthropod-borne diseases, such as louping-ill in sheep and Russian tick-borne encephalitis in dogs and horses, share the features of neurotropism, Purkinje cell necrosis, and a similar anatomic distribution with BoAstV-NeuroS1 infection.

Further research will be required to determine whether development of the neurologic signs seen here required other factors, including co-infections and/or a genetic or acquired immunodeficiency. PCR testing and genomic analysis of bovine fecal isolates also may provide information about the incidence and duration of virus shedding, which—as for other asymptomatic intestinal astrovirus infections—is expected to be short. In our study, in situ hybridization was negative on intestinal sections of the affected cattle. Seroprevalence studies in healthy cattle of different ages could measure any prior exposure to BoAstV-NeuroS1 and determine how frequently such asymptomatic infections occur.
